# Genome-wide profiling of miRNA-gene regulatory networks in mouse postnatal heart development—implications for cardiac regeneration

**DOI:** 10.3389/fcvm.2023.1148618

**Published:** 2023-05-22

**Authors:** Umesh Chaudhari, Lotta Pohjolainen, Heikki Ruskoaho, Virpi Talman

**Affiliations:** Drug Research Program and Division of Pharmacology and Pharmacotherapy, Faculty of Pharmacy, University of Helsinki, Helsinki, Finland

**Keywords:** miRNAs, gene regulatory networks, transcriptomics, neonatal mouse heart, cardiac development, cardiac regeneration

## Abstract

**Background:**

After birth, mammalian cardiomyocytes substantially lose proliferative capacity with a concomitant switch from glycolytic to oxidative mitochondrial energy metabolism. Micro-RNAs (miRNAs) regulate gene expression and thus control various cellular processes. Their roles in the postnatal loss of cardiac regeneration are however still largely unclear. Here, we aimed to identify miRNA-gene regulatory networks in the neonatal heart to uncover role of miRNAs in regulation of cell cycle and metabolism.

**Methods and results:**

We performed global miRNA expression profiling using total RNA extracted from mouse ventricular tissue samples collected on postnatal day 1 (P01), P04, P09, and P23. We used the miRWalk database to predict the potential target genes of differentially expressed miRNAs and our previously published mRNA transcriptomics data to identify verified target genes that showed a concomitant differential expression in the neonatal heart. We then analyzed the biological functions of the identified miRNA-gene regulatory networks using enriched Gene Ontology (GO) and KEGG pathway analyses. Altogether 46 miRNAs were differentially expressed in the distinct stages of neonatal heart development. For twenty miRNAs, up- or downregulation took place within the first 9 postnatal days thus correlating temporally with the loss of cardiac regeneration. Importantly, for several miRNAs, including miR-150-5p, miR-484, and miR-210-3p there are no previous reports about their role in cardiac development or disease. The miRNA-gene regulatory networks of upregulated miRNAs negatively regulated biological processes and KEGG pathways related to cell proliferation, while downregulated miRNAs positively regulated biological processes and KEGG pathways associated with activation of mitochondrial metabolism and developmental hypertrophic growth.

**Conclusion:**

This study reports miRNAs and miRNA-gene regulatory networks with no previously described role in cardiac development or disease. These findings may help in elucidating regulatory mechanism of cardiac regeneration and in the development of regenerative therapies.

## 1. Introduction

Mammalian heart development is a complex and tightly regulated process, which involves cell differentiation, proliferation, migration, growth, and maturation. Soon after birth, mammalian cardiomyocytes (CMs) terminally differentiate and lose proliferation capacity permanently. Very few CMs retain the capacity of proliferation throughout the life and less than 1% of CMs renew annually in humans (1, 2). Myocardial infarction results in irreversible damage to heart muscle leading to loss of as many as 1 billion CMs (3). Due to the negligible regeneration capacity, lost CMs cannot be replaced by new CMs in the advent of injury such as myocardial infarction. Cardiac muscle damage therefore leads to cardiac remodeling, which results in cardiac dysfunction and progressive heart failure.

Interestingly, certain fishes like zebrafish, and amphibians like newts and axolotls retain a complete cardiac regeneration capacity throughout their life (4). Moreover, 1-day old neonatal mouse heart possesses full regenerative potential after cardiac injury (5). However, this regenerative potential rapidly diminishes after birth and is completely lost by the end of the first week of life due to CM cell cycle withdrawal. While losing proliferative capacity, neonatal CMs undergo metabolic switch from glucose metabolism to fatty acid oxidation due to increased oxygen tension and higher levels of circulating fatty acids after birth (6). Many studies have demonstrated the significant interconnection between CM proliferation and mitochondrial metabolism (7, 8). Therefore, in-depth understanding of molecular mechanisms of metabolism-mediated cell cycle regulation is crucial to develop strategies to promote endogenous cardiac regeneration (9–13).

Small non-coding microRNAs (miRNAs) are ∼18–24 nucleotides long and bind to complementary sequences on messenger RNA (mRNA) to negatively regulate gene expression either by inducing degradation or by inhibiting translation of mRNA into proteins (14, 15). MiRNAs exhibit regulatory functions in a variety of biological and pathological processes, such as regulation of cell cycle, proliferation, differentiation, energy metabolism, apoptosis, stress response, and immune response. Recently, miRNAs have been shown to play a role in cardiac development (16, 17), diseases (18, 19), and regeneration (20, 21). However, the role of miRNAs in the regulation of CM proliferation and cell metabolism in human and other mammals remains unclear.

Currently, no clinical therapies are available to promote endogenous regeneration after cardiac injury. In quest of identifying therapeutic targets for endogenous cardiac regeneration, we explored miRNA dysregulation in mouse neonatal heart development with global miRNA expression profiling. Using miRNA-mRNA data integration we verified the potential target genes for the identified miRNAs, and then used the target genes in GO and KEGG pathway enrichment analyses to construct a miRNA-gene regulatory network. Our results illustrate the central role of miRNAs in neonatal heart development. We identified several miRNAs, for which there is no previously described role in cardiac biology, as potential regulators of cardiac regeneration.

## 2. Materials and methods

### 2.1. Preparation of ventricular tissue samples

**Figure 1:**
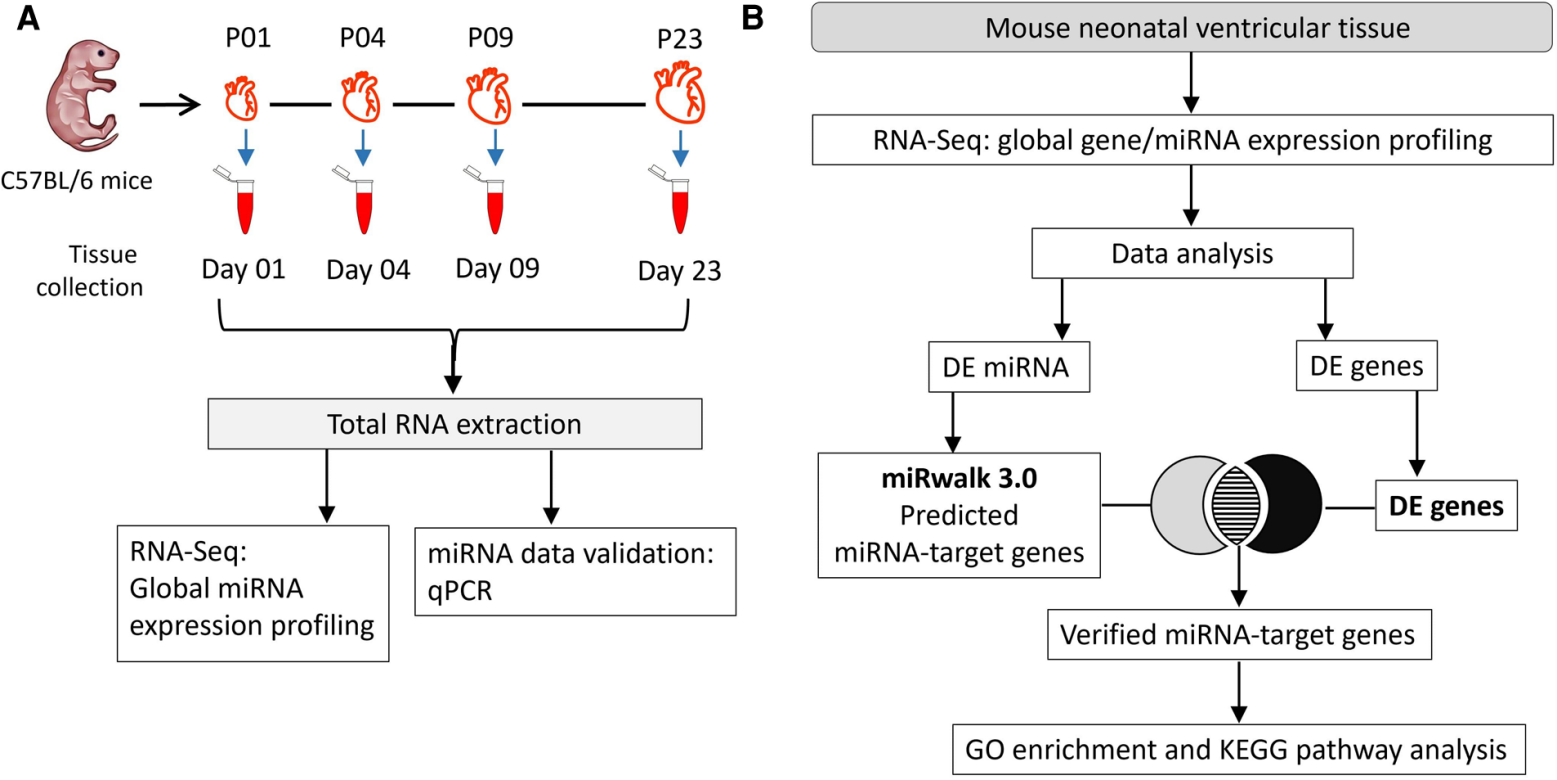
Experimental workflow. (**A**) Total RNA was collected on postnatal days 1, 4, 9, and 23 (P01, P04, P09 and P23, respectively) from mouse ventricles. RNA samples from 4 hearts/group were used in global miRNA expression profiling and data validation studies using qPCR. (**B**) Flow chart of data analysis and integration of miRNA sequencing data with transcriptomic data from (23) to identify target genes. Putative gene targets of miRNAs were verified from differentially regulated genes identified in our previous transcriptomic data analysis (23). DE, differentially expressed; FC, fold change.

Mouse ventricular tissue (VT) samples were collected at four postnatal heart development time points as shown in the Figure 1A. Male and female mouse pups (strain C57BL/6JOlaHsd) were obtained from the University of Helsinki Laboratory Animal Center. A total of 16 pups (4 per group) were sacrificed by decapitation on postnatal days 1, 4, 9 and 23 (P01, P04, P09 and P23, respectively) before weaning. All animals were housed and terminated in accordance with the 3R principles of the EU directive 2010/63/EU governing the care and use of experimental animals and following local laws and regulations. The use of animals for collecting tissues was reviewed and approved by Laboratory Animal Center of HiLIFE, Helsinki Institute of Life Sciences, University of Helsinki (internal license KEK20-012), as defined in the EU directive 2010/63/EU. For P01, P04 and P09 samples, pups were fed on mother's milk only, while P23 pups were given access to general mouse diet in addition to mother's milk. Harvested ventricles were cut in small pieces (4–8 pieces for P01, P04 and P09 samples; several pieces for P23), rinsed in phosphate-buffered saline, snap frozen in liquid nitrogen, and then stored at −80°C.

### 2.2. Isolation and culture of neonatal mouse cardiac cells

Neonatal mouse ventricular cells were isolated from 1-day old C57BL/6JOlaHsd mice. Twenty-five mice in total were sacrificed by decapitation. Ventricles were excised and dissociated into single-cell suspensions by combining mechanical and enzymatic dissociation using Neonatal Heart Dissociation Kit mouse and rat (Miltenyi Biotec) according to the manufacturer's instructions. CM and non-cardiomyocyte (non-CM; mainly fibroblasts and endothelial cells) populations were isolated using magnetic labeling by Neonatal Cardiomyocyte Isolation Kit mouse (Miltenyi Biotec) according to manufacturer's instructions. Isolated cells were plated on gelatin-coated 12-well plates at 400,000–500,000 cells/well in DMEM/F12 medium supplemented with 10% fetal bovine serum (FBS), 100 U/ml penicillin and 100 µg/ml streptomycin. On the following day, the plating medium of CMs was changed to complete serum free medium (DMEM/F12 supplemented with 2.5 mg/ml bovine serum albumin, 100 units/ml penicillin, 100 µg/ml streptomycin, 5 µg/ml insulin, 5 μg/ml transferrin, 5 ng/ml selenium, 2.8 mM sodium pyruvate, and 0.1 nM triiodothyronine) and plating medium of non-CMs was changed to fresh plating medium. Cells were grown for further four days before RNA sample preparation.

### 2.3. Total RNA extraction and purification

To ensure efficient tissue homogenization and high-quality total RNA extraction, VT samples were homogenized in TriZol® reagent (Thermo Fisher Scientific) using 1 mm zirconia beads and FastPrep-24 5G instrument (MP Biomedicals) with the following settings: speed 6.0 m/s, adapter Quick Prep, time 30s, cycles 4, rest time 300s. Cultured cells were homogenized by lysing in Trizol® reagent. Chloroform was added to homogenized samples and the homogenates were allowed to separate into 3 different layers. RNA in the upper aqueous layer was precipitated using isopropanol, washed with 75% ethanol and redissolved in RNase-free water. Extracted total RNA was purified using DNA-free Kit (Ambion) according to manufacturer's instructions. Integrity and quality of the purified RNA was analyzed using NanoDrop™ 2000 Spectrophotometer and Agilent 4200 TapeStation system.

### 2.4. Small RNA sequencing and data analysis

Small RNA (smRNA) sequencing libraries were prepared using 500 ng of total RNA from each sample and using the SMARTer smRNA-Seq kit for Illumina (Takara Bio) following manufacturer's recommended standard protocol. Briefly, smRNA libraries were generated by step-by-step processes viz. polyadenylation, complementary DNA (cDNA) synthesis, and polymerase chain reaction (PCR) based amplification. Next generation sequencing (NGS) of smRNA libraries (loading concentration 1.5 pM) was performed on an Illumina NextSeq 500 system. The sequencing reads were demultiplexed with Illumina Bcl2Fastq software, and raw data was trimmed using Trimmomatic tool. Data was aligned with mouse genome (GRCm 38.p6) using STAR tool. FeatureCounts program from Subread package was then used to count reads aligning to genes. Differential expression of smRNAs was determined by applying DEseq2 package in R. The data were then filtered for miRNAs and transcripts with very low expression levels (average expression counts <300 counts for all time points) were excluded from further analysis. Statistically signiﬁcant differential expression was deﬁned as fold change >1.8 and FDR-adjusted *p* < 0.01.

### 2.5. miRNA-target gene prediction and verification of miRNA-target genes

Potential target genes of differentially expressed miRNAs were obtained using the miRWalk 3.0 database (22). To verify target genes, the miRWalk-predicted target genes that were differentially expressed genes (fold change ≥1.8; FDR-adjusted *p* ≤ 0.01) in our previously published transcriptomic data (23) were identified and named “verified target genes”. Predicted target genes of upregulated miRNAs were verified using the list of downregulated genes and vice versa. The data analysis workflow is depicted in Figure 1B.

### 2.6. Gene ontology enrichment and KEGG pathway analysis

Database for Annotation, Visualization, and Integrated Discovery (DAVID) was employed in Gene Ontology (GO) and Kyoto Encyclopedia of Genes and Genomes (KEGG) pathway enrichment analysis (24). Enriched GO: Biological Process (GO:BP), GO: Cellular Components (GO:CC), and KEGG pathways with *p*-value ≤ 0.05 were identified based on the verified miRNA-target genes and were used to construct miRNA-gene regulatory network. MiRNAs, for which we were able to verify less than 40 differentially expressed target genes based on the miRWalk prediction and mRNA transcriptomics data were not used in GO and KEGG pathway enrichment analysis.

### 2.7. Quantitative real-time PCR

Quantitative real-time PCR (qPCR) was used to validate differential expression of selected miRNAs in VT samples and to compare expression levels in VT, CMs and non-CMs. The cDNA synthesis from total RNA was performed using miRCURY LNA RT kit (Qiagen) according to the manufacturer's protocol. cDNA was diluted 1:80 with nuclease free water as per manufacturer's instructions and then used as a template. qPCR was performed in 384-well plate format using miRCURY LNA miRNA custom PCR panel (Qiagen). The custom panel contained 18 target miRNAs, 4 reference miRNAs (U6 snRNA, miR-130a-3p, 5S rRNA, and miR-28-5p), and 2 RNA spike-ins (UniSp3 and UniSp6) as quality controls. MiRNA amplification was preformed using validated primers (Qiagen), miRCURY SYBR® Green Master Mix (Qiagen), and nuclease-free water (Qiagen) on LightCycler 480® real-time PCR instrument (Roche) as per manufacturer's PCR cycling conditions. Quantification values (Cq) of miRNAs were determined using Roche LightCycler 480® system software. Relative miRNA expression level was determined by analyzing Cq values of miRNAs using the *ΔΔ*Ct method. Statistical analysis was done using *Δ*Ct values and two-tailed Student's t-test method. *p*-value <0.05 was considered as statistically significant.

## 3. Results

### 3.1. miRNA profiling identified differentially regulated miRNAs at different stages in neonatal heart development

**Figure 2:**
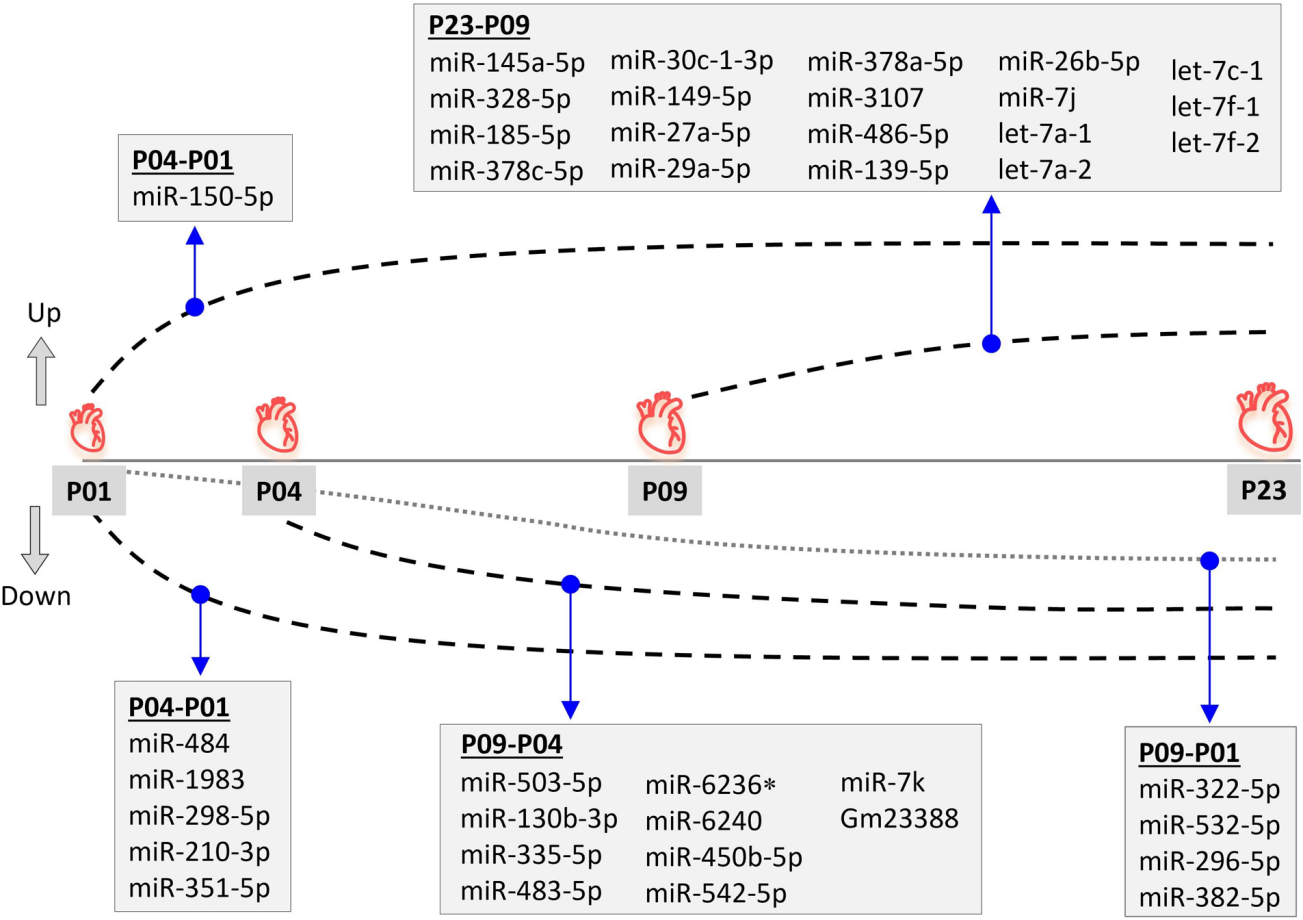
Differential miRNA expression pattern during neonatal mouse heart development. Differentially expressed (fold change ≥1.8; FDR-adjusted *p* < 0.01) miRNAs are shown for time-point comparisons: P04–P01, P09–P04, P09–P01, and P23–P09. All miRNAs apart from miR-6236 remained up- or downregulated also in later time point comparisons, but are only listed for the first time point comparison in which differential expression was observed. *miR-6236 was downregulated in the P09-P01 comparison, but not in P23-P01 comparison.

**Figure 3:**
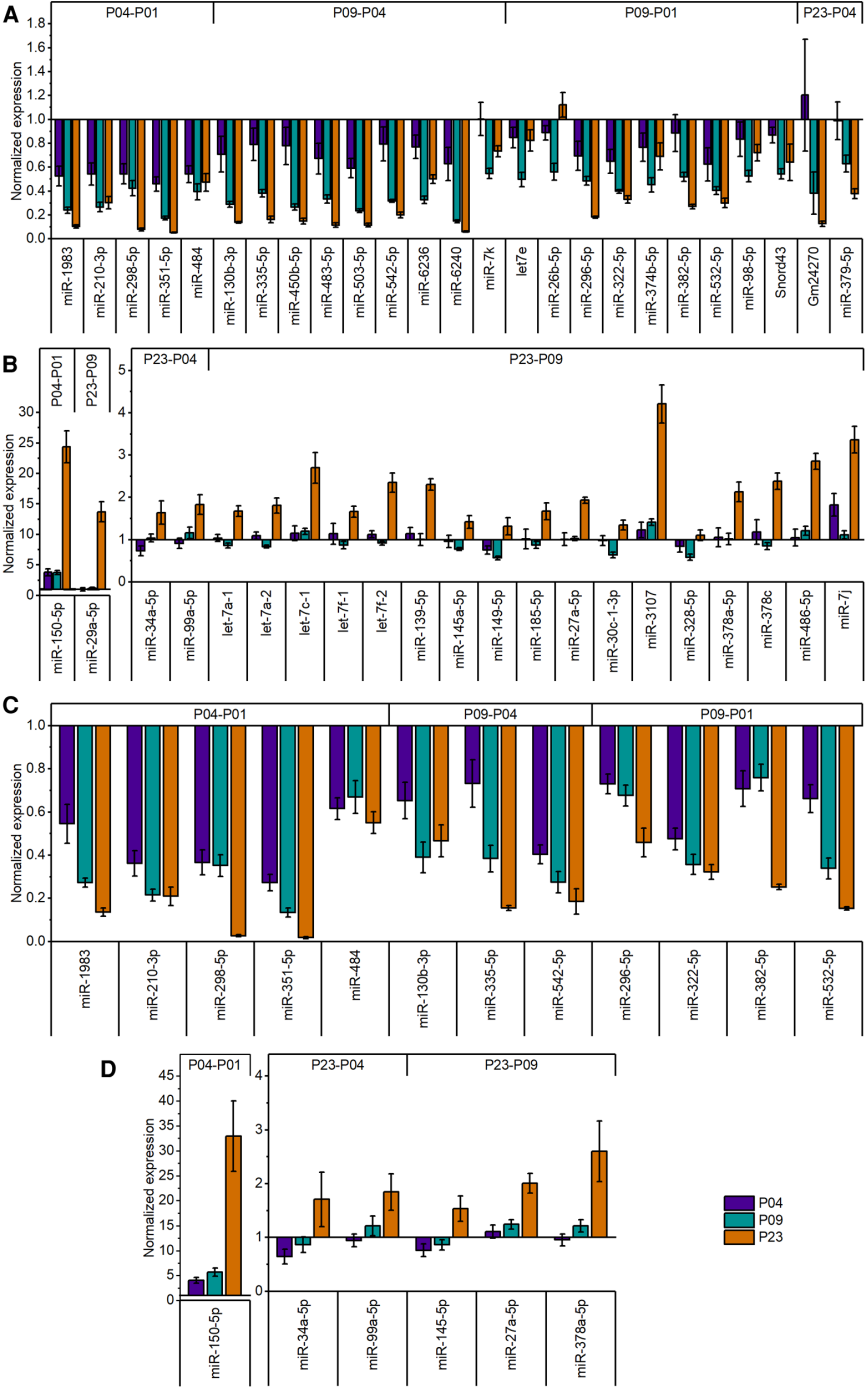
Relative expression levels of differentially expressed miRNAs in postnatal mouse heart. (**A**) Expression levels of downregulated (fold change ≥1.8; FDR-adjusted *p* < 0.01) miRNAs in global miRNA profiling. (**B**) Expression levels of upregulated (fold change ≥1.8; FDR-adjusted *p* < 0.01) miRNAs in global miRNA profiling. The data are presented as normalized to P01 (mean ± STDEV; *n* = 4 animals). (**C,D**) validation of miRNA-sequencing results by qPCR for downregulated (**C**) and upregulated (**D**) miRNAs. Top 18 differentially expressed miRNAs from different time points were selected for validation. Cq values were normalized to the average value of two housekeeping miRNAs (miR-130a-3p and miR-28-5p) from the same sample. The data are shown as normalized to P01 (mean ± STDEV; *n* = 4 animals).

Since miRNAs are central regulators of gene expression, and since our previous multi-omics study (23) did not provide information on small non-coding RNAs that may regulate the transcriptomic, proteomic and metabolomic changes in neonatal mouse hearts, we sought to determine the expression levels of miRNAs in mouse ventricular tissue over the neonatal period. We collected ventricular tissue RNA samples at time points corresponding to our previous study (P01, P04, P09 and P23) and quantified the expression of small RNAs by smRNA NGS. We then filtered the data for miRNAs and analyzed changes in miRNA expression levels by comparing various time points from P01 to P23. We outlined time-dependent expression patterns of consistently deregulated miRNAs after comparing P04–P01, P09–P04, and P23–P09 groups (Figure 2). The relative expression levels of differentially expressed miRNAs in all time point comparisons are shown in Figures 3A,B. In the P04–P01 comparison, we found six differentially expressed miRNAs: one upregulated (miR-150-5p) and five downregulated (miR-484, miR-1983 miR-298-5p, miR-210-3p and miR-351-5p). These 6 miRNAs showed consistent up/downregulation in all time points after P01. In the P09–P04 comparison, we identified 13 downregulated miRNAs, of which 10 were not downregulated in the P04–P01 comparison, but no new upregulated miRNAs (Figures 2, 3A,B). The downregulated miRNAs were also consistently downregulated from P04 to P23 with the exception of miR-6236, which was only downregulated in the P09-P04 comparison. In the P23–P09 comparison, we observed upregulation of 19 miRNAs that were not upregulated in previous time points (Figures 2, 3A,B).

We also compared the time point P09 (neonatal heart with no regenerative capacity) to P01 (neonatal heart with full regenerative capacity) and identified 24 differentially expressed miRNAs (Supplementary File S1). Nine of these were miRNAs that had not been differentially expressed in previous comparisons, and 4 showed consistent downregulation also in the P23–P01 comparison (Figure 2). The remaining 5 miRNAs showed lowest expression levels at P09 but thereafter their expression increased so that their expression level at P23 was close to that at P01 (miR-374b, let-7e, miR-98, Snord43, and miR-26b-5p) or significantly upregulated when compared to P01 (miR-26b-5p). List of differentially expressed miRNAs from all time-point comparisons is provided in the Supplementary File S1.

We then selected several of the top differentially expressed miRNAs for validation by qPCR. The results confirmed the significant downregulation in all downregulated miRNAs selected for validation (Figure 3). Similarly, qPCR confirmed the increase of miRNA expression levels of upregulated miRNAs selected for validation (Figure 3).

### 3.2. Relative miRNA expression in ventricular tissue, cardiomyocytes, and non-cardiomyocytes

**Figure 4:**
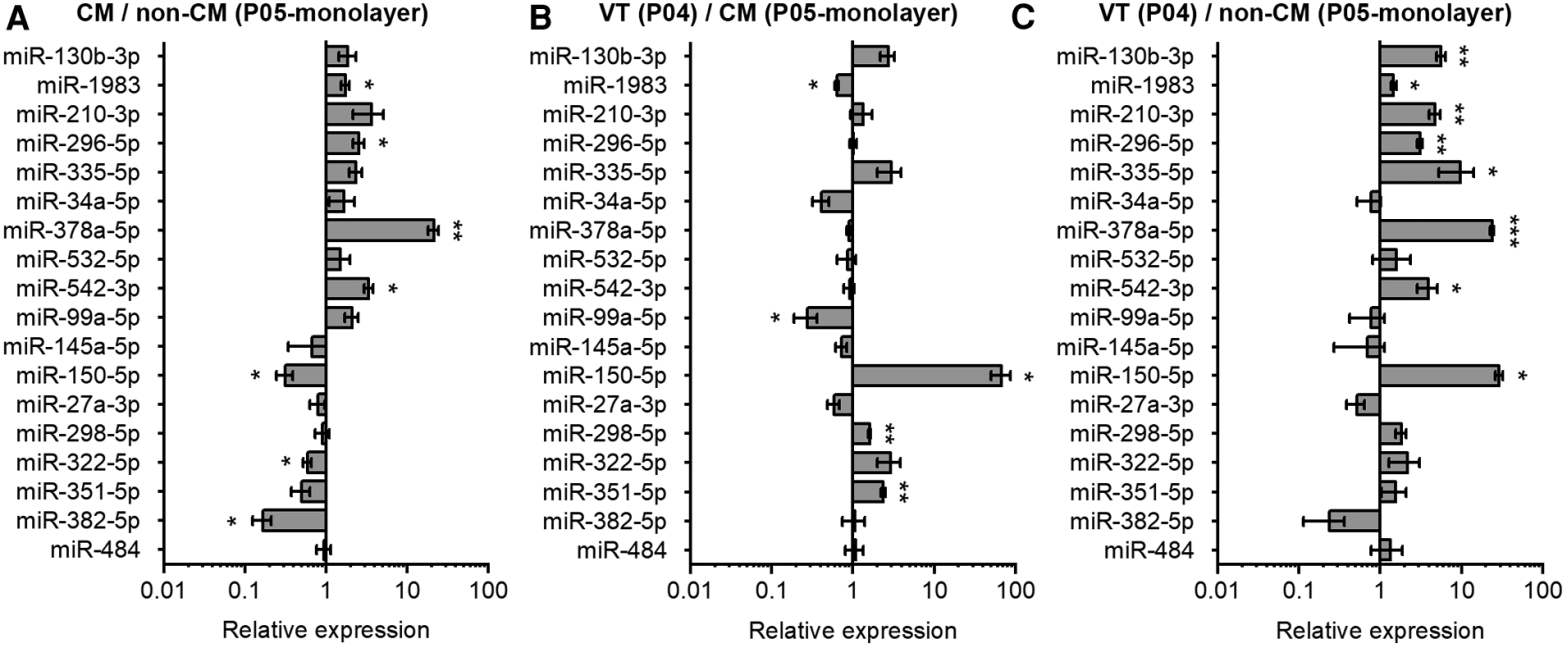
Comparison of miRNA expression in neonatal mouse cardiomyocytes, non-cardiomyocytes and ventricular tissue. The expression of miRNAs in mouse heart tissue and isolated cells was analyzed using qPCR and results are shown as relative miRNA expression for three comparisons: (**A**) cardiomyocytes (CM) vs. non-cardiomyocytes (non-CM); (**B**) ventricular tissue (VT) vs. CM; and (**C**) VT vs. non-CM. The data are presented as average ± standard error of the mean; *n* = 3. *p ≤ 0.05, **p ≤ 0.005, ***p ≤ 0.0005.

In addition to CMs, the VT samples used for the miRNA analysis also contain non-CMs. We therefore investigated the relative expression of selected miRNAs in CMs and non-CMs. Data in Figure 4A demonstrates higher expression of miR-378a-5p, miR-542-5p, and miR-296-5p, and lower expression miR-382-5p and miR-150-5p in CMs compared to non-CMs. We further compared the miRNA expression levels in VT samples to *in vitro* monolayer cultures of CMs and non-CMs. miR-351-5p and miR-150-5p showed significantly higher expression in VT samples compared to CM monolayer culture, while miR-99a-5p showed lower expression in VT than in cultured CMs (Figure 4B). On the other hand, the expression of miR-150-5p, miR-378a-5p, miR-335-5p, miR-130b-3p, miR-210-3p, miR-542-5p, and miR-296-5p was higher in VT samples than in non-CM monolayer culture (Figure 4C). The discrepancies between the CM/non-CM and tissue vs. *in vitro* comparisons can be attributed to effects of cell isolation and *in vitro* conditions to miRNA expression as well as differences in survival or proliferation rates of specific cell types during cell isolation and the 4-day *in vitro* monolayer culture.

### 3.3. Construction of miRNA-gene regulatory networks

After identification of differentially regulated miRNAs, we used miRWalk 3.0 to predict the candidate target genes for each differentially expressed miRNA and verified the predicted target genes by integrative analysis with our previously published mRNA transcriptomic data (23). A comprehensive list of verified target genes of each miRNA is provided in the Supplementary File S2. We then performed GO and KEGG pathway enrichment analysis to investigate sequential changes in neonatal heart development. Significantly enriched GO and KEGG pathways, specifically associated with cell cycle progression, cell proliferation, maturation (including cell growth, mitochondrial maturation, cytoskeletal organization and metabolic reprograming), and relevant regulatory networks were used to construct miRNA-gene regulatory networks.

### 3.4. miRNA-gene regulatory networks in early neonatal heart development (P01 to P04)

**Figure 5:**
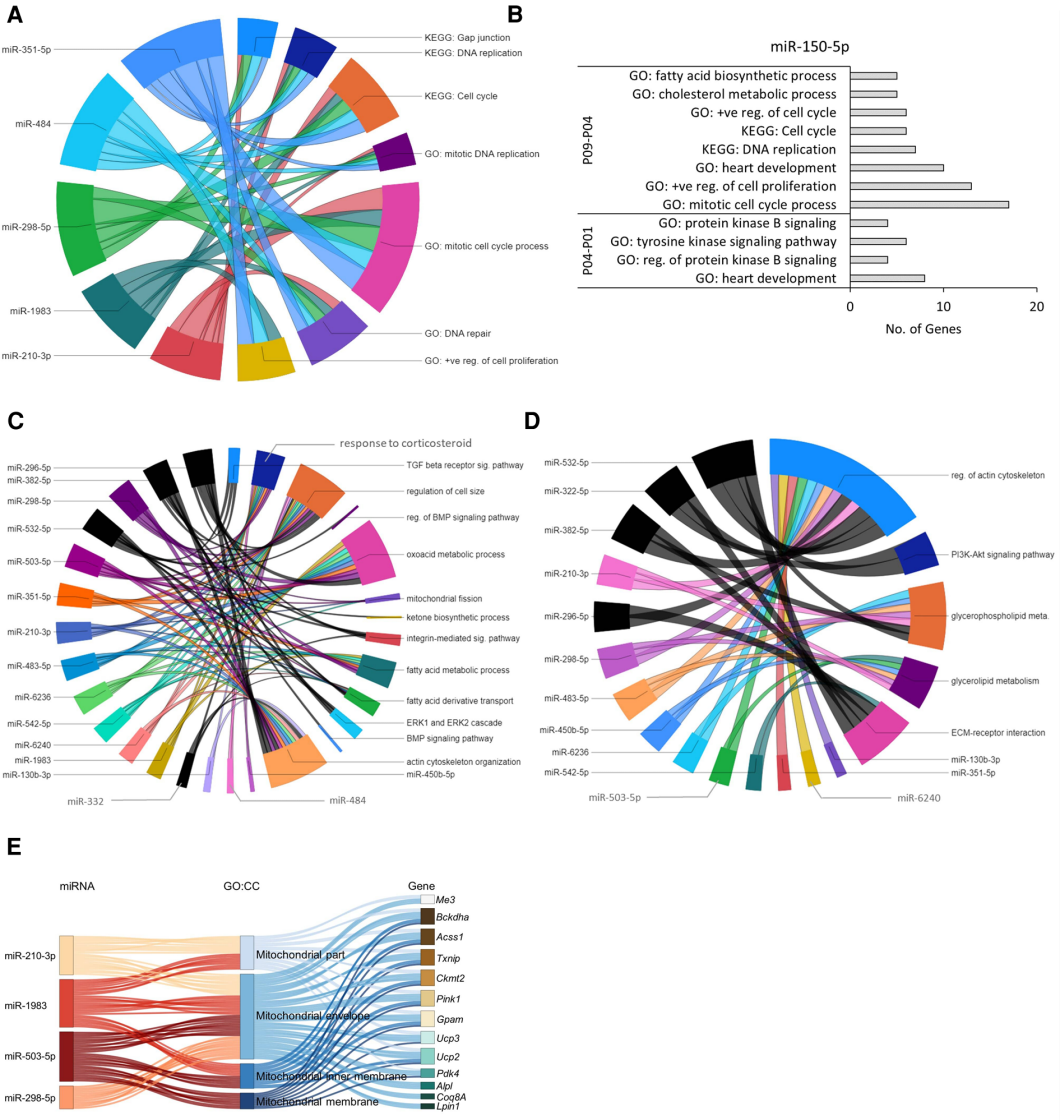
Predicted miRNA-gene regulatory networks in mouse hearts within the first 9 postnatal days. (**A**) Chord diagram showing that target genes of early downregulated miRNAs (P04–P01) were enriched in GO:BP and KEGG pathways (*p* ≤ 0.05) associated with cell cycle and proliferation. (**B**) The upregulated miR-150-5p showed negative regulatory effect on GO biological processes and KEGG pathways (*p* ≤ 0.05) related to cell cycle and proliferation in P01 to P09 time span. (**C,D**) Chord diagram demonstrating interaction network between enriched biological processes (**C**) and KEGG pathways (**D**) with downregulated miRNAs identified in P09–P04 and P09–P01 comparisons. The enrichment of the target genes of each miRNA with the GO biological processes and KEGG pathways (*p* ≤ 0.05) are presented with a single color. Gene-regulatory networks of miRNAs from P09–P01 comparison are shown in black color. The size of the section representing each GO term or KEGG pathway is proportional to the number of target genes associated with the corresponding GO term or KEGG pathway. (**E**) Sankey plot showing mitochondria-associated GO:CC enriched in verified target genes of downregulated miRNAs in the P09–P04 comparison.

The gene regulatory network of the 5 miRNAs downregulated in the early neonatal phase from P01 to P04 included genes involved in mitotic cell cycle processes; DNA replication; DNA repair; and positive regulation of cell proliferation (Figure 5A). These upregulated biological processes associated with cell cycle events are in line with the short term proliferation potential of postnatal CMs and the switch from hyperplastic to hypertrophic growth within a week after birth (25). Upregulated DNA repair process is also in accordance with increase in oxidative DNA damage and DNA damage response in the CMs within the first week after birth (26).

In the P04–P01 comparison, the biological processes enriched in the target genes of the upregulated miR-150-5p include protein kinase B signaling; tyrosine kinase signaling pathway; and heart development (Figure 5B), suggesting early postnatal inhibition of AKT signaling, which is a key regulator of glucose metabolism and CM cell cycle progression (9). Furthermore, after P04 (P09–P04 comparison), biological processes and KEGG pathways associated with cholesterol metabolic process, mitotic cell cycle, DNA replication, positive regulation of cell proliferation, and heart development were enriched in the downregulated target genes of miR-150-5p (Figure 5B), suggesting a suppressive role for miR-150-5p in these processes. This data agrees with the previous findings showing a decrease in cell cycle processes and proliferation potential in neonatal CMs within the first week after birth (25). Furthermore, our observation that cholesterol metabolic processes are inhibited in this proliferative to non-proliferative transition is in line with numerous previous reports linking dysregulated cholesterol metabolism to cancer cell proliferation and survival (see for example 27, 28). Information about all enriched GO:BP and KEGG pathways (*p*-value ≤0.05) by verified target genes of each miRNA is provided in the Supplementary File S3.

### 3.5. miRNA-gene regulatory networks in the transition phase (P04 to P09) from proliferative to non-proliferative state in neonatal heart

The biological processes enriched in target genes of downregulated miRNAs in the P09–P04 comparison were related to response to corticosteroids; mitochondrial fission; fatty acid metabolic process; oxoacid metabolic process; actin cytoskeleton organization; and regulation of cell size (Figure 5C) and KEGG pathways regulation of actin cytoskeleton; ECM-receptor interaction; and glycerolipid metabolism (Figure 5D). Moreover, GO:CC enrichment analysis of target genes of downregulated miRNAs (miR-210-3p, miR-298-5p, miR-1983, and miR-503-5p) showed interaction with upregulated mitochondrial genes (Figure 5E). This is in line with the increase in mitochondrial mass and cytoskeletal maturation during early neonatal heart development (29, 30). Information about all enriched GO:BP, GO:CC, and KEGG pathways by each miRNA-target gene is provided in the Supplementary Files S4, S5.

### 3.6. miRNA-gene regulatory networks in maturation and hypertrophic growth phase of cardiac development (P09 to P23)

**Figure 6:**
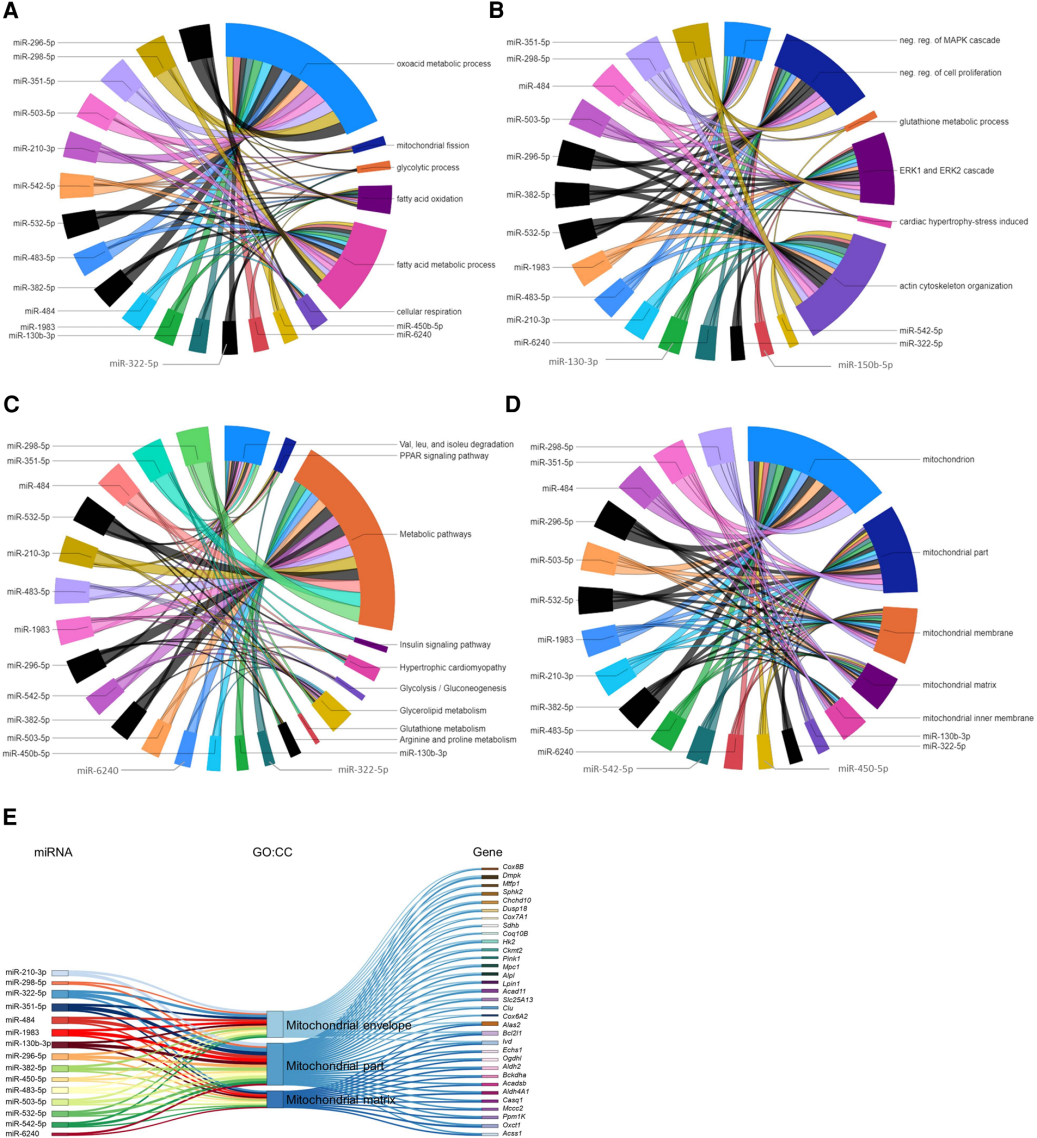
Gene regulatory networks of downregulated miRNAs after postnatal day 9 and miRNAs associated with mitochondrial genes. (**A,B**) GO biological processes with enriched verified target genes of miRNAs that were downregulated in the P23–P09 comparison. The data has been divided into two chord diagrams for clarity of presentation. (**C**) KEGG pathways enriched by verified target genes of miRNAs downregulated in the P23–P09 comparison. (**A–C**) The enrichment of the target genes of each miRNA with the GO biological processes and KEGG pathways (*p* ≤ 0.05) are presented with a single color in the chord diagrams. The size of the section representing each GO term or KEGG pathway is proportional to the number of target genes associated with the corresponding GO term or KEGG pathway. (**D**) Chord diagram demonstrating that verified target genes of miRNAs enriched in mitochondria-associated GO cellular components. The enrichment of the target genes of each miRNA with the GO cellular component are presented with a single color. Gene-regulatory networks of miRNAs from P09–P01 comparison are shown in black color. (**E**) Sankey plot showing mitochondria-associated GO:CC enriched in verified target genes of downregulated miRNAs in the P23–P09 comparison.

The ten miRNAs downregulated in the P09–P04 comparison showed consistent downregulation in post P09 heart development with the exception of miR-6236. Downregulation of these miRNAs removed inhibitory effect on the mRNA expression of genes in P09 to P23 period. The miRNA-gene regulatory networks showed positive regulatory effect on biological processes including oxoacid metabolic process; fatty acid metabolic process; fatty acid oxidation; glycolytic process; mitochondrial fission; and cellular respiration (Figure 6A). In addition, these miRNAs showed interaction with signaling processes, particularly, ERK1 and ERK2 cascade; actin cytoskeleton organization; stress-induced cardiac hypertrophy; and negative regulation of MAPK cascade and cell proliferation (Figure 6B). Furthermore, these miRNA-gene interaction activated KEGG pathways including valine, leucine, and isoleucine degradation; arginine and proline metabolism; and hypertrophic cardiomyopathy (Figure 6C). We also carried out GO:CC enrichment analysis and found that the target genes of downregulated miRNAs show enrichment of mitochondrial genes (Figures 6D,E; Supplementary File S8). These results are in line with mammalian neonatal CMs exiting cell cycle and undergoing structural, functional, metabolic, and mitochondrial maturation one week after birth (31, 32). The total number of mitochondrial genes targeted by each miRNA are shown in the Supplementary Figure S1. All enriched GO:BP and GO:CC terms from each downregulated miRNA are provided in the Supplementary Files S7, S8.

**Figure 7:**
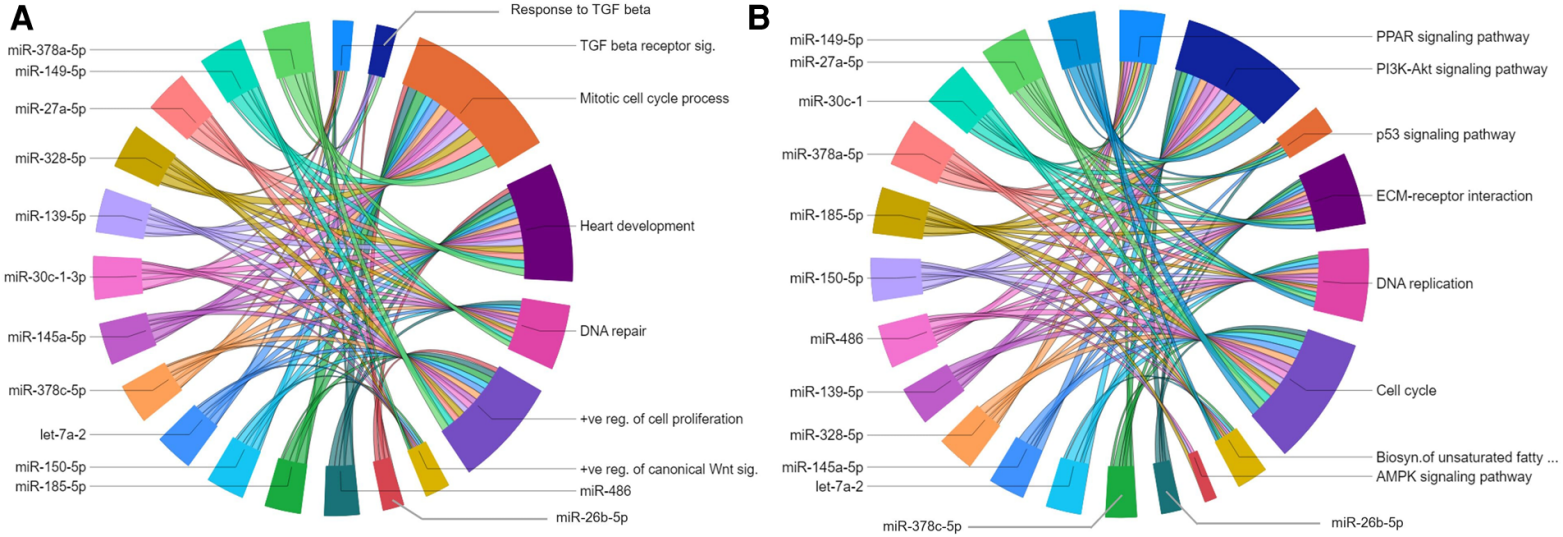
Gene regulatory networks of upregulated miRNAs after postnatal day 9. Chord diagrams showing negative regulation of GO biological processes (**A**) and KEGG pathways (**B**) by target genes of upregulated miRNAs. The enrichment of the target genes of each miRNA with the GO biological processes and KEGG pathways (*p* ≤ 0.05) are presented with a single color in the chord diagrams. The size of the section representing each GO term or KEGG pathway is proportional to the number of target genes associated with the corresponding GO term or KEGG pathway.

The gene regulatory networks of miRNAs upregulated in the P23–P09 comparison showed inhibitory effect on biological processes including mitotic cell cycle process; heart development; DNA repair; and positive regulation of cell proliferation (Figure 7A). Moreover, they suppressed gene expression linked to multiple KEGG pathways, such as PI3K-AKT signaling pathway; cell cycle; DNA replication; MAPK signaling pathway; PPAR signaling pathway; and ECM-receptor interaction (Figure 7B). Our results are consistent with the preceding findings that support the inhibition of cell cycle/proliferation related processes and signaling pathways towards the terminal differentiation of CMs (33, 34).

Of specific interest for cardiovascular development is the let-7 family of miRNAs, which has been shown to play a role in heart development and cardiovascular diseases (35). Of the five let-7 family members upregulated in our dataset, the miRwalk database provided predicted targets genes only for let-7a-1 and let7a-2. Since there were <40 target genes for let-7a-2, further analyses were only carried out with let-7a-2, target genes of which were linked to biological processes and KEGG pathways similar to other upregulated miRNAs in the P23–P09 comparison. List of all enriched GOs for each upregulated miRNA is provided in the Supplementary File S9.

### 3.7. miRNA-gene regulatory networks during the first 9 postnatal days

The additional P09–P01 comparison showed involvement of more miRNAs in neonatal heart development. The target genes of consistently downregulated miRNAs (miR-296-5p, miR-382-5p, miR-532-5p, and miR-322) in the P09–P01 comparison showed enrichment of biological processes associated with integrin-mediated signaling pathway and ERK1 and ERK2 cascade, and the KEGG pathway ECM-receptor interaction and regulation of actin cytoskeleton (Figures 5C,D). These results agree with the previous studies that reported the involvement of integrins in terminal differentiation of CMs and ERK1/2 activation induced cardiac hypertrophy (36, 37). Detailed information about all enriched biological processes and KEGG pathways by verified target genes of each miRNA obtained in P09–P01 comparison is provided in the Supplementary File S6.

### 3.8. Comparison to previous studies

Finally, to compare our findings to previous studies, which have reported miRNA expression patterns in neonatal heart (38, 39) or the effects of miRNAs on CM proliferation (40), we searched our sequencing data for miRNAs reported in these studies. Of the five miRNAs that Deng et al. (38) identified as upregulated in neonatal vs. adult rat hearts, we detected miR-15b and miR-708 expression in the mouse ventricular samples. While both miRNAs were downregulated with increasing age, miR-708 expression levels were below our threshold to be included in further analysis, and miR-15b was only downregulated in the P23–P01 comparison based on our criteria (Supplementary Figure S2A). Liu et al. (39) reported differential expression of 64 miRNAs in neonatal mouse hearts (P01 compared to P06 or P07). Our sequencing detected 51 of these miRNAs, and those miRNAs, whose expression levels were above threshold to be included for further analysis, are shown in Supplementary Figure S2B. Our results are largely in line with Liu et al.: we detected downregulation of miR-130b, miR-1983, miR-351, miR-450b, miR-483, miR-532 and miR-542 with increasing age in the neonatal period (time points up to P09 in our samples). In addition, miR-99b-3p was downregulated in the P23–P01 comparison, and there was a trend towards downregulation of miR-342-5p. However, let-7a-1 and miR-145 were upregulated in our data from P09 to P23 in contrary to the report by Liu et al. (39).

Furthermore, functional screening of human miRNAs on neonatal rodent CM proliferation by Eulalio et al. (40) identified a number of miRNAs affecting CM proliferation. The miRNAs with expression levels above our threshold to be included for further analysis are shown in Supplementary Figure S2C. We did not detect any members of the pro-proliferative miRNA families miR-518 and miR-302 in the mouse ventricular samples. The upregulation of several let-7 miRNAs at P23 reported in our study is in line with their antiproliferative role reported by Eulalio and co-workers. The expression levels of miR-23b, miR-130a, which were reported as pro-proliferative miRNAs in the screening carried out by Eulalio et al., were not differentially regulated in our data (Supplementary Figure S2C). However, the downregulation of miR-98 at P09 compared to P01 detected in this study and upregulation of miR-99a in the P23–P04 comparison (Supplementary Figure S2C) are contradictory to their effects on cell proliferation in the study by Eulalio et al.: overexpression of miR-98 inhibited CM proliferation and overexpression of miR-99a promoted CM proliferation (40). The other pro-proliferative miRNAs, identified by Eulalio et al., including miR-590-3p and miR-199a-3p, which promoted cardiac regeneration *in vivo* (40) were not detected (miR-590-3p) or were expressed at a level below the inclusion threshold of our analysis (miR-199a-3p).

## 4. Discussion

Immediately after birth, neonatal mammalian CMs undergo dramatic and rapid changes that include permanent cell cycle exit and a metabolic switch from glycolysis to fatty acid oxidation. The unresponsiveness of adult CMs to mitotic stimulation is a major hurdle in cardiac regeneration after cardiac injury (41–43). Genomic level studies are urgently required to find promising therapeutic targets to induce endogenous cardiac regeneration. MicroRNAs represent a group of such potential therapeutic targets (44). By regulating the expression levels of several genes, one miRNA may be able to regulate various processes that are involved in regeneration. In the present study, we determined differentially expressed miRNAs and constructed miRNA-gene regulatory networks in the neonatal mouse heart to identify new potential regulators of the postnatal loss of cardiac regeneration.

### 4.1. Early postnatal stage (P01 to P04)

Differential expression of the six miRNAs (miR-150-5p, miR-484, miR-1983, miR-298-5p, miR-210-3p, and miR-351-5p) from P01 to all later time points suggests a key role for these miRNAs in heart development. A previous study has shown that miR-298-5p and miR-351-5p are more highly expressed in the early prenatal stages of fetal heart development and are downregulated in the final stage of prenatal mouse heart development (45). In addition, downregulation of miR-1983 and miR-351-5p has been reported in mouse hearts from P01 to P06 and P07, respectively (39). To our knowledge, there are no published reports suggesting the involvement of miR-150-5p, miR-484, and miR-210-3p in mouse heart development.

In line with the dramatic decrease in CM proliferation after the first days of postnatal life, our miRNA-gene regulatory network model predicted that in early neonatal heart development (from P01 to P04) the upregulated miR-150-5p mediates negative regulation of cell cycle, DNA replication, and protein kinase B (AKT) signaling. In line with the prediction of our model, previous studies have linked miR-150-5p to AKT signaling (46) and suggested that it plays a tumor-suppressing role in various cancers (47–49). Consistent with our results, dual inhibition of MAPK and PI3K-AKT pathways using small molecules was recently reported to decrease proliferation and enhance maturation of human induced pluripotent stem cell-derived cardiomyocytes (hiPSC-CMs) (33). PI3K-AKT signaling is also a central regulator of glucose metabolism. Negative correlation of miR-150-5p expression with glucose utilization has been reported in the neonatal rat CMs (50). In our study the verified target genes of miR-150-5p did not however show enrichment with biological processes or KEGG pathways associated with glucose metabolism. On the other hand, our miRNA-gene regulatory network predicted an inhibitory effect on cholesterol metabolic process for miR-150-5p. Cholesterol biosynthesis is essential for mevalonate pathway and cell proliferation. Previous studies that have demonstrated the importance of mevalonate pathway in promoting proliferation of hiPSC-CMs and adult CMs *in vivo* in mice supports our prediction (23, 51). Based on these observations, we speculate that miR-150-5p may play a central role in CM proliferation.

During the first postnatal week, mouse CMs permanently exit cell cycle to cease proliferation. One of the mechanisms regulating this postnatal cell cycle exit is oxidative stress-induced DNA damage response due to the rapid increase in oxygen availability after birth (26). In line with these changes, the miRNA-gene regulatory network for the five miRNAs that were downregulated from P01 to P04 and remained downregulated at later time points (miR-298-5p, miR-351-5p, miR-484, miR-1983, and miR-210-3p) predicted a role for these miRNAs in the regulation of cell cycle, proliferation and DNA repair processes in the P01 to P04 time span. A recent study demonstrated that downregulation of miR-351-5p inhibited proliferation, while its overexpression promoted proliferation in mouse skeletal muscle (52). Similarly, miR-210-3p has been reported to protect H9c2 cardiomyoblasts from hypoxia-induced injury (53) and to regulate glioblastoma cell proliferation under hypoxic conditions (54). There are conflicting reports about the role of miR-484 in cell proliferation in cancer models (55, 56), and to our knowledge, a role in cell proliferation has not been described for miR-298-5p or miR-1983. Our model also predicted a role for miR-351-5p in the regulation of fatty acid oxidation and hypertrophy related processes in the heart. There are no previous reports supporting this prediction.

### 4.2. Postnatal transition from hyperplastic to hypertrophic growth (P04 to P09)

In addition to the five downregulated miRNAs from the P04–P01 comparison, which continued to be downregulated from P04 to P09, our study identified nine miRNAs that were downregulated in the P09–P04 comparison and remained downregulated to P23 (Figure 2). Moreover, one miRNA (miR-6236) was transiently downregulated in the P09–P04 comparison only. Of these, miR-483, miR-130, and miR-450b were found to be downregulated in a previous study between postnatal days 3 and 5 in mouse heart (57). To our knowledge, changes in the expression levels of the other seven miRNAs in neonatal mouse heart have not been reported. Our miRNA-gene regulatory network model predicted that these ten miRNAs play a role in the regulation of cell cycle and proliferation. No mechanistic studies are currently available that highlight the relationship between these miRNAs in neonatal cardiac development. However, in a previous study, overexpression of a mimic of miR-6240, one of the downregulated miRNAs in our data, induced proliferation in hiPSC-CMs (57).

The miRNA-gene regulatory networks for these miRNAs (excluding miR-130b-3p) predicted involvement in processes such as fatty acid and glycerolipid metabolism, regulation of cell size, and regulation of actin cytoskeleton, which suggests potential roles in postnatal CM maturation for these miRNAs. Postnatal CMs are known to undergo maturation that includes cytoarchitectural and metabolic maturation, mitochondrial biogenesis, and developmental hypertrophy (58–60). In particular, for miR-322-5p, miR-532-5p, miR-296-5p, and miR-382-5p, the miRNA-gene regulatory networks predicted involvement in the regulation of integrin mediated signaling pathway and ECM-receptor interaction. Our predicted results are in line with the previous report suggesting involvement of integrins in cardiac hypertrophy (61).

The five downregulated miRNAs that we identified in the P04–P01 comparison were also associated with upregulated target genes in the P04 to P09 period. According to the GO and KEGG pathway analysis, these target genes are involved in developmental hypertrophic growth and mitochondrial maturation. The downregulated miRNAs in the P09–P04 comparison were also predicted to interact with mitochondrial genes, which we verified using mRNA transcriptomics data. Supporting our findings, previous studies have reported the involvement of miR-210, miR-484 and miR-130b-3p in the regulation of mitochondrial metabolism in CMs and non-CMs (62–67). The biological functions predicted by our miRNA-gene regulatory network model are thus in line with the well-known postnatal transition from hyperplastic to hypertrophic growth and maturation of mitochondria to allow increased oxidative metabolism.

In the additional P09–P01 comparison, four miRNAs (miR-322-5p, miR-532-5p, miR-296-5p and miR-382-5p) were downregulated and remained downregulated at P23. Of these, only miR-532-5p has been suggested to play a role in mouse heart development during the fetal period (45). There are no reports describing differential expression or possible biological roles for the three other miRNAs in heart development.

### 4.3. Changes after the regenerative window (P09 to P23)

Upon comparison of time points after the regenerative window (P23–P09), we detected upregulation 19 miRNAs that were not differentially expressed in earlier time points. Of these miRNAs, miR-486, miR-3107, and miR-378a have been reported to exhibit a similar trend of upregulation during neonatal mouse heart development (57) and the expression of miR-145, miR-378, miR-30c, miR-27a, miR-29a, miR-26a has been reported in the adult mouse heart (45). For the 11 remaining miRNAs, we did not find information about their expression levels in the heart.

Our miRNA-gene regulatory networks predicted negative regulation of GO and KEGG pathways essential for cell cycle and proliferation. In consistence with our results, overexpression of miR-29a has been reported to inhibit CM proliferation (45). There is no reported role for the other 18 miRNAs on CM proliferation. However, in alignment with our prediction, these miRNAs negatively regulate cell proliferation in various cancer cell types (68–79). Upregulation of these miRNAs might thus represent a major hurdle in reactivation of CM proliferation following a cardiac injury.

Our data shows upregulation of the let-7 family members in the maturation and growth phase of CMs (P09 to P23). While target gene predictions for all let-7 miRNA family members are not available, the verified target genes of let-7a-2 showed enrichment of biological processes and KEGG pathways involved in promoting cell cycle and proliferation, suggesting an antiproliferative role for let-7a-2 in the mouse heart. The previously reported involvement of the let-7 miRNA family in the proliferation of neonatal rat CMs (40) and maturation and adult-like metabolism of hiPSC-CMs supports our prediction (80).

## 5. Summary and conclusions

In summary, our work identified 46 miRNAs, which were differentially expressed at different stages of neonatal heart development. For several of these miRNAs there is no previously described role in cardiac development or disease. Twenty miRNAs were differentially expressed during the time when the heart loses its regenerative capacity, and the miRNA-gene regulatory networks suggest that several of these miRNAs play a role in regulating processes linked to cardiac regeneration. Importantly, most of the identified miRNAs are conserved in humans, which not only supports the hypothesis that they are important regulators of processes common to all mammalian species, but also highlights the translation potential of these findings. As the present study was limited to bioinformatics-based modelling of potential roles for the miRNAs, further studies are needed to assess the exact roles of these miRNAs in different cardiac cell types, heart development and regeneration.

## Data Availability

The datasets generated for this study can be found in the NCBI Gene Expression Omnibus (http://www.ncbi.nlm.nih.gov/geo, accession number GSE221538). All other data are available from the corresponding author upon reasonable request.
