# Supplementary material for: Genome-wide profiling of miRNA-gene regulatory networks in mouse postnatal heart development—implications for cardiac regeneration
Source: Front Cardiovasc Med. 2023 May 22;10:1148618. doi: 10.3389/fcvm.2023.1148618 (PMC10241105; doi:10.3389/fcvm.2023.1148618)
Supplement: Supplementary file 10 [file Datasheet1.pdf]

Supplementary Material

1 List of Additional Supplementary Files

Supplementary Figure S1: Image 1
Supplementary Figure S2: Image 2
Supplementary File S1: Table 1
Supplementary File S2: Table 2
Supplementary File S3: Table 3
Supplementary File S4: Table 4
Supplementary File S5: Table 5
Supplementary File S6: Table 6
Supplementary File S7: Table 7
Supplementary File S8: Table 8
Supplementary File S9: Table 9

2 Figure Legends for Supplementary Figures

Supplementary Figure S1. Differentially expressed miRNAs regulate expression of mitochondrial genes in the neonatal mouse heart. The graph shows the total number of mitochondrial genes identified from the verified target genes for each miRNA. Target genes were first predicted using miRWalk 3.0 and then verified using mRNA transcriptomics data (Talman et al. 2018) as described in the Methods section. The verified target genes were used in the GO enrichment analysis for cellular component. Time point comparisons P09-P04 (postnatal day 9 vs postnatal day 4), P09-P01 and P23-P09 are shown in yellow, red and black, respectively.

Supplementary Figure S2. Expression patterns of selected miRNAs from previous studies. Relative miRNA expression in mouse ventricular tissue at 1, 4, 9 and 23 days after birth (P01, P04, P09 and P23, respectively) based on smRNA sequencing. The miRNAs were selected from the following previously published studies: Deng et al. 2017 (A); Liu et al. 2014 (B) and Eulalio et al. 2012 (C). The data are normalized to P01 and are shown as mean + stdev; n=4 animals for each group.

3 References

Deng S, Zhao Q, Zhen L, Zhang C, Liu C, Wang G, Zhang L, Bao L, Lu Y, Meng L, Lü J, Yu P, Lin X, Zhang Y, Chen YH, Fan H, Cho WC, Liu Z, Yu Z. Neonatal Heart-Enriched miR-708 Promotes Proliferation and Stress Resistance of Cardiomyocytes in Rodents. Theranostics (2017) 7:1953-65.

Eulalio A, Mano M, Dal Ferro M, Zentilin L, Sinagra G, Zacchigna S, Giacca M. Functional

screening identifies miRNAs inducing cardiac regeneration. Nature (2012) 492:376-81.

Liu HL, Zhu JG, Liu YQ, Fan ZG, Zhu C, Qian LM. Identification of the microRNA expression profile in the regenerative neonatal mouse heart by deep sequencing. Cell Biochem Biophys (2014) 70:635-42.

Talman V, Teppo J, Poho P, Movahedi P, Vaikkinen A, Karhu ST, Trošt K, Suvitaival T, Heikkonen J, Pahikkala T, Kotiaho T, Kostiainen R, Varjosalo M, Ruskoaho H. Molecular Atlas of Postnatal Mouse Heart Development. J Am Hear Assoc (2018) 7:e010378.
