# Supplementary figures and images for: Genome-wide profiling of miRNA-gene regulatory networks in mouse postnatal heart development—implications for cardiac regeneration
Source: Front Cardiovasc Med. 2023 May 22;10:1148618. doi: 10.3389/fcvm.2023.1148618 (PMC10241105; doi:10.3389/fcvm.2023.1148618)

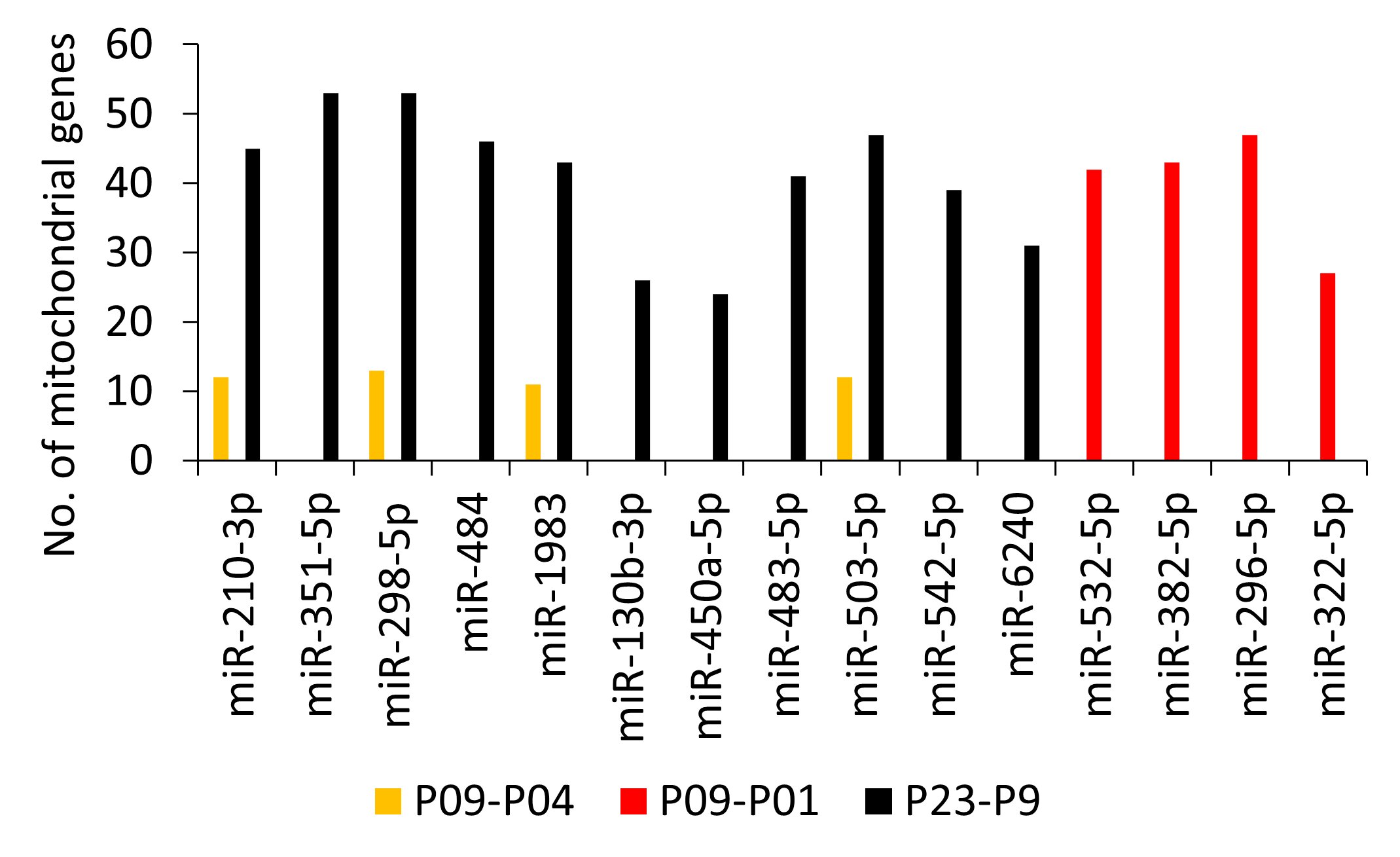

Supplement: Supplementary file 11 [file Image1.jpeg]

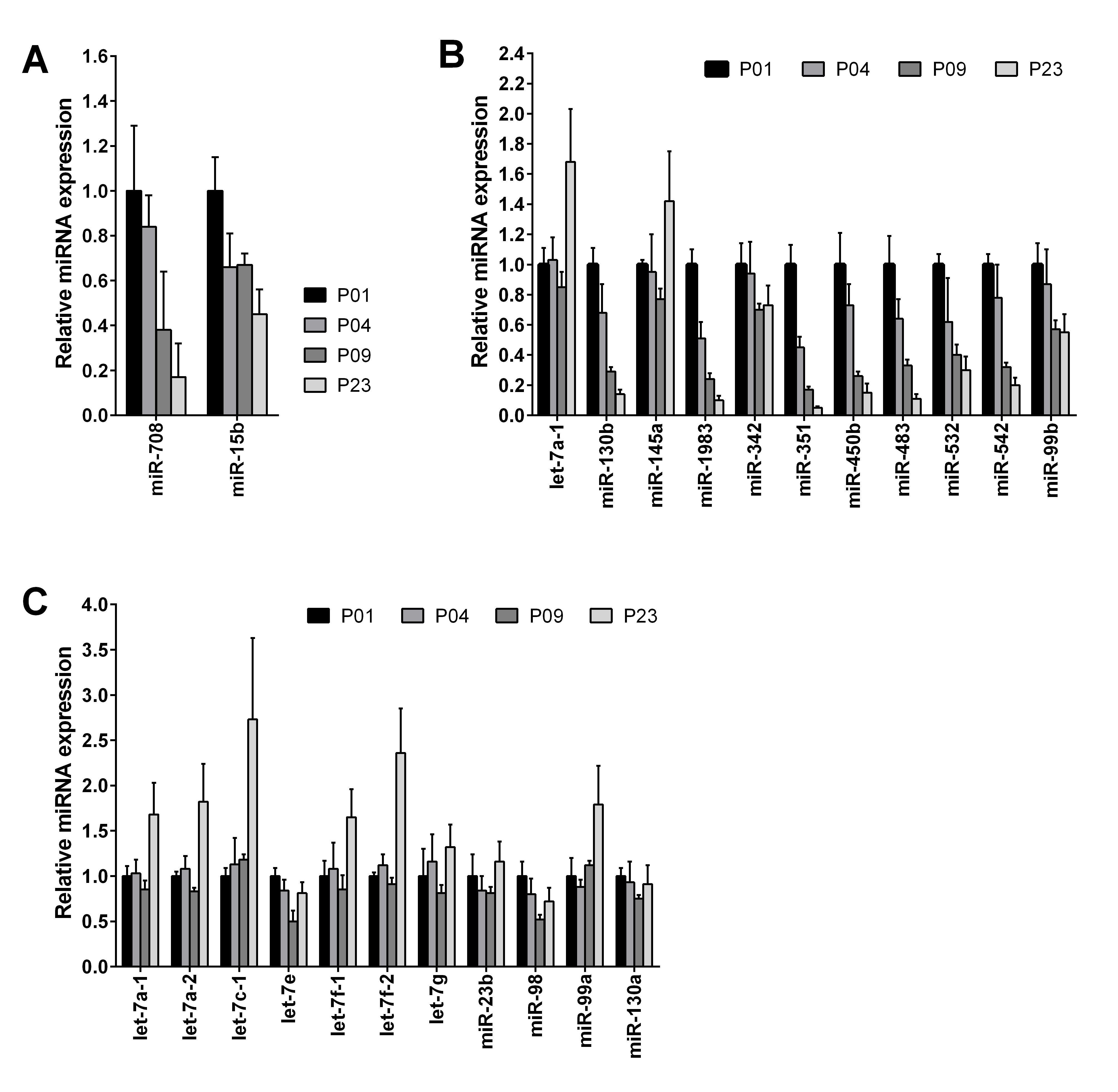

Supplement: Supplementary file 12 [file Image2.jpeg]
